# Flos Puerariae Extract Ameliorates Cognitive Impairment in Streptozotocin-Induced Diabetic Mice

**DOI:** 10.1155/2015/873243

**Published:** 2015-04-28

**Authors:** Zhong-he Liu, Hong-guang Chen, Pan-feng Wu, Qing Yao, Hong-ke Cheng, Wei Yu, Chao Liu

**Affiliations:** ^1^Xianning Hospital of Traditional Chinese Medicine, Xianning 437100, China; ^2^Hubei Province Key Laboratory on Cardiovascular, Cerebrovascular, and Metabolic Disorders, Hubei University of Science and Technology, Xianning 437100, China

## Abstract

*Objective*. The effects of Flos Puerariae extract (FPE) on cognitive impairment associated with diabetes were assessed in C57BL/6J mice. *Methods*. Experimental diabetic mice model was induced by one injection of 50 mg/kg streptozotocin (STZ) for 5 days consecutively. FPE was orally administrated at the dosages of 50, 100, or 200 mg/kg/day, respectively. The learning and memory ability was assessed by Morris water maze test. Body weight, blood glucose, free fatty acid (FFA) and total cholesterol (TCH) in serum, malondialdehyde (MDA), superoxide dismutase (SOD), catalase (CAT), glutathione peroxidase (GSH-Px), and acetylcholinesterase (AChE) activities in cerebral cortex and hippocampus were also measured. *Results*. Oral administration of FPE significantly improved cognitive deficits in STZ-induced diabetic mice. FPE treatment also maintained body weight and ameliorated hyperglycemia and dyslipidemia in diabetic mice. Additionally, decreased MDA level, enhanced CAT, and GSH-Px activities in cerebral cortex or hippocampus, as well as alleviated AChE activity in cerebral cortex, were found in diabetic mice supplemented with FPE. *Conclusion*. This study suggests that FPE ameliorates memory deficits in experimental diabetic mice, at least partly through the normalization of metabolic abnormalities, ameliorated oxidative stress, and AChE activity in brain.

## 1. Introduction

There are around 366 million people worldwide with diabetes mellitus. And this number is estimated to reach 552 million by 2030 [1]. Diabetes can lead to various micro- and macrovasculopathy affecting kidneys, eyes, heart, and nervous system. Although peripheral neuropathy is the most common of the complications associated with long-term diabetes mellitus, the central nervous system (CNS) is also impaired. Evidence from larger epidemiological studies has demonstrated that diabetes has detrimental effects on cognitive functions and may increase the risk of dementia and the progression to diabetes represents a state of accelerated aging [2–4].

Diabetes is a complex of metabolic disorder termed as metabolic syndrome, which includes a lot of factors such as hyperglycemia, hyperinsulinemia, dyslipidemia. Those factors act in concert with each other and contribute to the various complications of diabetes. Previous studies have shown that oxidative stress is involved in the pathogenesis of diabetes and its complications. The increased reactive oxygen species (ROS) derived from hyperglycemia or hyperlipidemia is considered to be the most important contributor in the development and the progression of diabetes [5]. Oxidative stress is also regarded as the etiological factor in the development of neurodegenerative diseases [6]. Excessive ROS production or antioxidant deficiency causes cognitive impairment and morphological abnormalities in different brain regions. Treatment with antioxidants has been shown to protect neurons against diabetes-induced excitotoxicity and neurodegenerative conditions [7].

Flos Puerariae is the dry bud of* Pueraria lobata* (Willd.) Ohwi, a plant in the genus* Pueraria* of the pea family Fabaceae, subfamily Faboideae. Different active constituents extracted from Flos Puerariae have proved to exert activities such as hypoglycemic, hypolipidemic antioxidant, antidiabetic, antithrombotic, and antiallergic activities [8, 9]. Previous studies in our lab have demonstrated that crude extract from Flos Puerariae (FPE) could improve cognitive deficits following acute ethanol intoxication in mice, which is likely through its antioxidant property [10]. It stimulated our interests to explore if FPE could exert similar effects in diabetes. In the present study, we have tried to investigate whether FPE has a protective effect against cognitive deficits and the underlying mechanisms in experimental diabetic mice.

## 2. Materials and Methods

### 2.1. Preparation of Flos Puerariae Extract (FPE)

The crude extract of Flos Puerariae was prepared at the Phytochemistry Laboratory, Department of Materia Medica, Hubei University of Science and Technology. The standard procedures of exaction were followed as we described before [11]. In brief, the dried aerial part of Flos Puerariae was mixed with 50% (v/v) methanol solution with the solid-to-liquid ratio at 1 : 30. The mixture was extracted with ultrasound for 2 h at 70°C. The extracted products were then purified sequentially by petroleum ether, ethanol, and chloroform-butyl alcohol and eluted gradually with mixed mobile phase of methanol-chloroform solution in the silica gel column system. In the end, the isolated ingredients were further analyzed by color reaction, ultraviolet spectrophotometry, high performance liquid chromatography, infrared spectrum, and mass spectrum. Total flavonoids in the final extract were proved to be 17.5%. And five primary isoflavones were identified as irisolidone, genistein, daidzein, kakkalide, and puerarin.

### 2.2. Induction of Diabetic Model and Treatment with FPE

Male C57BL/6J mice (25 ± 2 g) were purchased from Laboratory Animal Center (Hunan, China). All animals were treated in accordance with the Guide for the Care and Use of Laboratory Animals published by the US National Institutes of Health (NIH Publication Number 85–23, revised 1996). Mice over 20 months old were used for the experiments. The diabetic model was set up by STZ 50 mg/kg injected intraperitoneally once a day for 5 consecutive days [12]. All mice fasted 10 hours prior to injection. 4 weeks after injection, the mice were tested for sufficient levels of hyperglycemia. Blood glucose level was assessed using hand-held glucometer (Changsha Sinocare Inc. China) by tail vein puncture blood sampling. Those blood glucose values <11.1 mmol/L were excluded from this experiment. Diabetic mice were randomly divided into 5 groups: control group, diabetic model (DM) group, and FPE groups (HFPE: high dose, 200 mg/kg; MFPE: medium dose, 100 mg/kg; LFPE: low dose, 50 mg/kg). FPE groups were orally administrated with FPE once a day for 10 weeks. The control and model groups were treated with equal volume of saline. There are twelve to fifteen mice in each group. All animals were provided with food and water* ad libitum*.

### 2.3. Morris Water Maze

Learning and memory performances were assessed at room temperature (23 ± 1°C) by Morris water maze test [13]. The water maze pool consisted of a circular water tank (diameter 100 cm, depth 30 cm) and a circular transparent platform (diameter 10 cm). The maze was conceptually divided into I, II, III, and IV, four equal quadrants by four poles along the perimeter of the pool. The platform was put under 1 cm of the water surface at the center of one quadrant. Every spatial sign around the maze was constantly kept during the testing period. Morris water maze test consists of two parts: (1) place navigation test. In each test session, mice were gently placed into water at the same point of one quadrant in turn. Mice were then allowed to find the platform and rest on it for 15 s. Those who failed to find the location within 90 s were gently guided to the platform and stay on it for 15 s. Each mouse was trained four times daily on four consecutive days. In the fifth day, the escape latency in searching the platform was recorded for analysis. (2) Spatial probe trial: after the place navigation test finished on the fifth day, the platform was removed from the water tank. And mice were allowed to swim freely for 90 s. The time was spent in the target quadrant where the platform located was recorded for analysis.

### 2.4. Biochemical Analysis

At the end of the experiment, mice were anesthetized and decapitated. Blood samples were collected and sera were separated and stored at −20°C until biochemical assay. The brain was then gently removed and cerebral cortex and hippocampus were separated on an ice-chilled glass plate. The tissues were homogenized in 10% (w/v) ice-cold saline and centrifuged at 3,000 r/min for 10–15 minutes. Supernatants were stored at −80°C until biochemical assay. Body weight and blood glucose measured by hand-held glucometer were monitored every week. Serum free fatty acid (FFA) and total cholesterol (TC) were determined by autobiochemical analysis system following the instruction of the detection kits. MDA, a by-product of lipid peroxidation, was measured by thiobarbituric acid reacting substances (TBARS) method [14]. SOD was determined based on its ability to inhibit the oxidation of oxymine by O_2_^−^ produced from the xanthine/xanthine oxidase system [15]. Catalase activity (CAT) was measured by employing hydrogen peroxide to generate H_2_O and O_2_ [16], and Glutathione peroxidase (GSH-Px) activity was determined using the procedure described by Armstrong and Browne [17]. And the activities of AChE were measured by colorimetry according to the corresponding commercial kits. All kits mentioned above are provided by Nanjing Jiancheng Institute of Biological Engineering (Nanjing, China). Protein concentration was determined by the Coomassie blue protein-binding assay [18] using bovine serum albumin (BSA) as a standard.

### 2.5. Statistical Analysis

Group differences in behavior tests were analyzed with two-way analysis of variance (ANOVA) followed by the Student-Newman-Keuls test for multiple comparisons among different groups. The other data were analyzed using one-way ANOVA followed by the Student-Newman-Keuls test. Differences were considered to be significant at *P* < 0.05.

## 3. Results

### 3.1. Effect of FPE on Learning and Memory Abilities in Experimental Diabetic Mice

**Figure 1:**
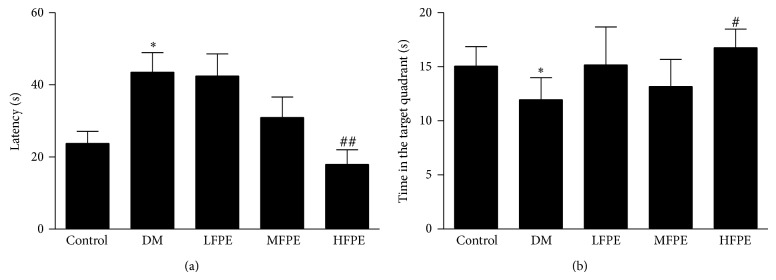
Effect of FPE on learning and memory abilities in experimental diabetic mice. (a) FPE-treated diabetic mice significantly reduced the prolonged escape latency in a dose dependent manner. (b) FPE-treated diabetic mice markedly increased the time searching in the target quadrant. LFPE: low dose, 50 mg/kg; MFPE: medium dose, 100 mg/kg; HFPE: high dose, 200 mg/kg. Data are means ± S.E.M. *n* = 12–15. ^∗^ *P* < 0.05 versus control group; ^∗∗^ *P* < 0.01 versus control group; ^#^ *P* < 0.05 versus DM group; ^##^ *P* < 0.01 versus DM group.

In Morris water maze, it took longer time to find the hidden platform for mice in model group than control in the place navigation test (*P* < 0.05). Administration of FPE in diabetic mice significantly reduced the prolonged escape latency in a dose dependent manner. (Figure 1(a), *P* < 0.01, HFPE versus model group). The spatial probe trial helps to determine whether the animal would take a spatial learning strategy to locate the platform in the target quadrant. As shown in Figure 1(b), the time was spent in the target quadrant where the platform located was greatly reduced in model group compared to control group (*P* < 0.05). FPE-treated diabetic mice markedly increased the time in searching the platform in the target quadrant (*P* < 0.05 HFPE versus model group).

### 3.2. FPE Decreased Blood Glucose and Normalized Body Weight in Experimental Diabetic Mice

**Figure 2:**
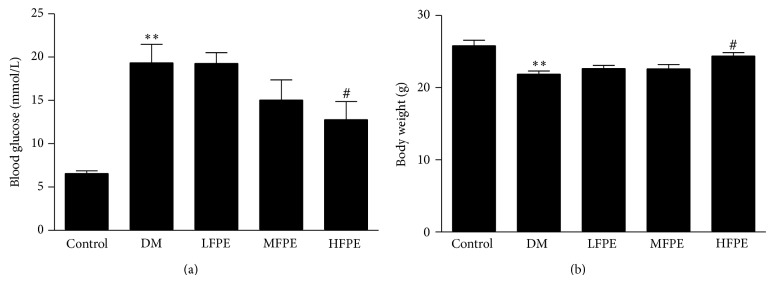
FPE decreased blood glucose and normalized body weight in experimental diabetic mice. (a) Application with FPE dose-dependently decreased blood glucose in experimental diabetic mice. (b) Application with FPE normalized body weight in the experimental diabetic mice. LFPE: low dose, 50 mg/kg; MFPE: medium dose, 100 mg/kg; HFPE: high dose, 200 mg/kg. Data are means ± S.E.M. *n* = 12–15. ^∗^ *P* < 0.05 versus control group; ^∗∗^ *P* < 0.01 versus control group; ^#^ *P* < 0.05 versus DM group; ^##^ *P* < 0.01 versus DM group.

As shown in Figures 2(a) and 2(b), the blood glucose significantly increased 4 weeks after injection with STZ in model group mice, while the body weight markedly decreased as compared with control group (*P* < 0.01). Application with FPE could dose-dependently decrease the blood glucose level and normalize the body weight in experimental diabetic mice (*P* < 0.05 HFPE versus model group).

### 3.3. Effect of FPE on Serum Free Fatty Acid (FFA) and Total Cholesterol (TCH) Levels in Experimental Diabetic Mice

**Figure 3:**
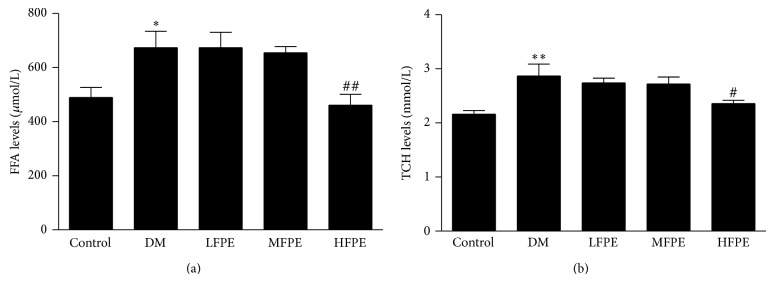
Effect of FPE on serum FFA and TCH levels in experimental diabetic mice. (a) Application with FPE significantly decreased serum FFA levels in experimental diabetic mice. (b) Application with FPE significantly decreased serum TCH levels in experimental diabetic mice. LFPE: low dose, 50 mg/kg; MFPE: medium dose, 100 mg/kg; HFPE: high dose, 200 mg/kg. Data are means ± S.E.M. *n* = 12–15. ^∗^ *P* < 0.05 versus control group; ^∗∗^ *P* < 0.01 versus control group; ^#^ *P* < 0.05 versus DM group; ^##^ *P* < 0.01 versus DM group.

As shown in Figures 3(a) and 3(b), the serum FFA and TCH content were significantly higher in model group than those in control group (*P* < 0.05 or *P* < 0.01). Compared to model group, serum FFA and TCH levels were markedly decreased in FPE-treated groups (*P* < 0.01 or *P* < 0.05 HFPE versus model group).

### 3.4. Effect of FPE on MDA Formation, Superoxide Dismutase (SOD), Catalase (CAT), and Glutathione Peroxidase (GSH-Px), and Acetylcholinesterase (AChE) Activities in Cerebral Cortex and Hippocampus of Experimental Diabetic Mice

**Figure 4:**
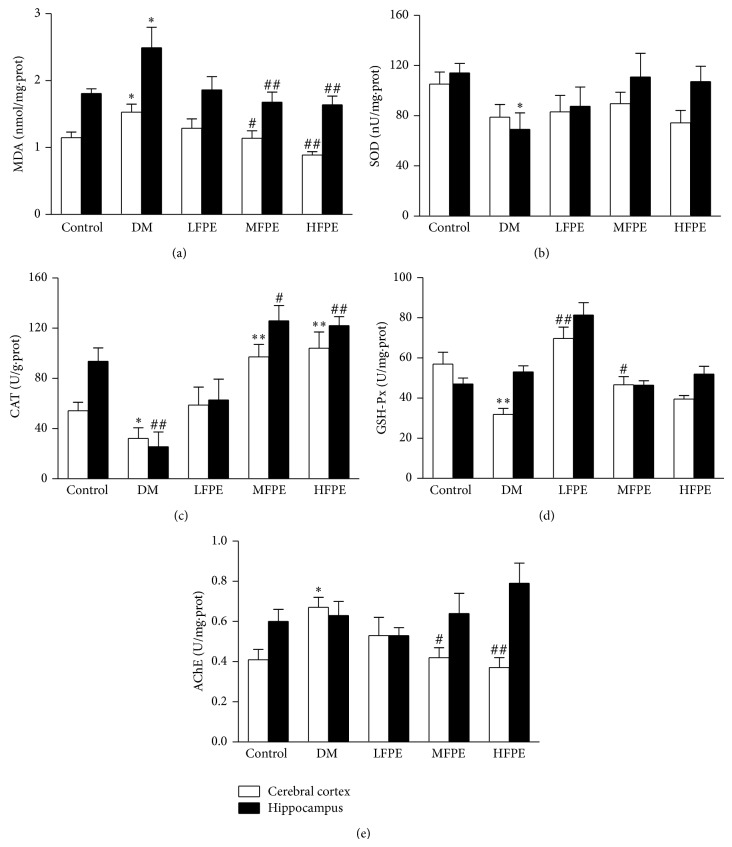
Effect of FPE on MDA formation, SOD, CAT, GSH-Px, and AChE activities in cerebral cortex and hippocampus of experimental diabetic mice. (a) FPE-treated diabetic mice significantly inhibited MDA level both in cerebral cortex and hippocampus of experimental diabetic mice. (b)–(d) Administration with FPE enhanced SOD, CAT and GSH-Px activity in cerebral cortex of experimental diabetic mice. (e) Treatment with FPE dose-dependently reversed AChE activity in cerebral cortex of experimental diabetic mice. LFPE: low dose, 50 mg/kg; MFPE: medium dose, 100 mg/kg; HFPE: high dose, 200 mg/kg. Data are means ± S.E.M. *n* = 12–15. ^∗^ *P* < 0.05 versus control group; ^∗∗^ *P* < 0.01 versus control group; ^#^ *P* < 0.05 versus DM group; ^##^ *P* < 0.01 versus DM group.

The oxidant-antioxidant status of brain was assessed by determining the levels of lipid peroxidation and antioxidant enzymes SOD, CAT, and GSH-Px activities both in cerebral cortex and hippocampus. As shown in Figures 4(a)–4(d), MDA formation was significantly increased in model group. In contrast, SOD, CAT, and GSH-Px activity was decreased as a compared model to control group (*P* < 0.05 or *P* < 0.01). And treatment with FPE significantly alleviated the MDA level both in cerebral cortex and hippocampus (*P* < 0.01 or *P* < 0.05), which was in parallel with enhanced CAT and GSH-Px activity in cerebral cortex or hippocampus (*P* < 0.01 or *P* < 0.05). SOD activity was also increased in hippocampus in PFE-treated groups. However, no significant difference has been detected between model group and PFE-treated groups. Additionally, AChE activity markedly increased in cerebral cortex of diabetic model mice. FPE administration dose-dependently reversed AChE activity compared to the model group (Figure 4(e), *P* < 0.05 or *P* < 0.01).

## 4. Discussion

Due to the increasing incidence of diabetes worldwide, extensive research is being performed to develop new antidiabetic agents and determine their mechanisms of action. Accordingly, a number of experimental diabetic animal models have been developed, of which rodent models are the most described. Streptozotocin (STZ) is well known to cause pancreatic *β*-cell damage and has been widely used to induce experimental diabetes in research [19]. STZ is transported into *β*-cells via the glucose transporter GLUT2 and causes DNA damage, which results in the fact that insulin-secreting cells undergo necrosis [20]. In the present study, we used low dose STZ injection for multiple times to induce the experimental diabetic model, which mimics the type 1 diabetes characterized by absolute deficiency of endogenous insulin secreting. Most of the mice that received STZ injection showed increased hyperglycemia in our experiments and coupled with other metabolic disturbance including increased serum free fatty acid and total cholesterol. These data indicated that the diabetic model had been established successfully and further confirmed that diabetes elicits a complex of metabolic disorder.

Insulin signal is very important for brain function. STZ injected intracerebroventricularly (icv) to inhibit the function of the neuronal insulin receptor can induce sporadic Alzheimer disease in animal [21]. Epidemiological studies have also demonstrated the association between diabetes and dementia [22]. People with diabetes are >1.5-fold more likely to develop AD. And 80% of Alzheimer's disease (AD) patients are present with diabetes or impaired fasting glucose [23]. The memory loss or cognitive decline in diabetic individuals may be caused by vascular and nonvascular factors, as those protein metabolism dysfunctions also contribute to the increased risk of AD in diabetes [24]. Diabetes and AD share several commonalities including impaired glucose metabolism, increased oxidative stress, insulin resistance, inflammation, and amyloidosis, contributing to the overlapping pathology and thereby compounding disease symptoms and progression [25]. The Morris water maze test is one of the most widely accepted models for the evaluation of spatial learning and memory in rodents. In this study, the retrieval of memory is conventionally evaluated by decreased time used for finding the hidden platform and an increase in the time spent in the target quadrant. The diabetic model group showed typical signs of learning and memory deterioration in that escape latency was prolonged and the time spent in the target quadrant was shorter in contrast to control mice. After FPE treatment, those mice showed a remarkable improvement in their learning and memory abilities. The antidementia effect of FPE in experimental diabetic mice was clearly demonstrated in our study. And this effect may be related to the ameliorated hyperglycemia and dyslipidemia by FPE treatment [26].

Hyperglycemia and the fatty acid oxidation pathway are major inducers of reactive oxygen species [27]. ROS production can be in equilibrium with the antioxidant capacity in cells. Antioxidant enzymes, such as SOD, CAT, and GSH-Px, and other nonenzymatic antioxidants, such as vitamins A, E, and C, are natural detoxification molecules that reduce or scavenge ROS. All of these antioxidant systems can decrease the potency of particular reactive species or render them completely harmless. Unfortunately, this antioxidant defense may not counteract the prooxidant effect developed in diabetes and its complications. Oxidative stress has also been considered as the main mechanism involved in the pathogenesis of neurodegenerative diseases. The brain is particularly susceptible to oxidation by reactive oxygen species (ROS), which may be due to its dependency on aerobic metabolism, large contents of polyunsaturated lipids in mitochondrial and cell plasma membranes, and low antioxidant defenses. In the present study, the compromised actives of SOD, CAT, and GSH-Px have been found in the diabetic mice. The production of MDA, the hallmark of lipid peroxidation, is increased in the cortex and hippocampus of model mice. In contrast, increased activities of antioxidant enzymes and reduced MDA in cortex and hippocampus have been shown to be supplemented with FPE. The antioxidant properties of FPE are consistent with our previous reports [10, 28].

In addition to antioxidant, cholinesterase inhibition has been another mainstay for the treatment of AD since cholinergic deficiency is the important etiology for AD. And most drugs approved and licensed for AD are AChE inhibitors [29]. Our data have shown that AChE activity was increased in the cortex of diabetic mice, which was reversed by FPE administration. This effect is probably attributed by antioxidant property of PFE. Evidences have shown that oxidative injury is one of the main reasons that eventually lead to degeneration or atrophy of cholinergic neurons in the basal forebrain [30]. Thus, scavenging of ROS may be capable of modulating glutamate excitotoxicity and prevent abnormities on cholinergic neurons [31].

It is known that the antioxidant bioactivity of medicinal plants is mostly attributable to their phenolic components, of those the most common is flavonoids [32]. There are a lot of bioactive components originated from Flos Puerariae. The crude extract from Flos Puerariae contains flavone glycosides, flavonoid C-glycosides, isoflavone glycoside, saponins, sterol glycoside, alkaloid, amino acids, and sugars. The total flavonoids have been confirmed more than 17.5% through our optimized extraction procure. Five primary isoflavones have been identified as irisolidone, genistein, daidzein, kakkalide, and puerarin. Each of them exhibited potent antioxidant capacity when tested in vitro. We have demonstrated that FPE prevents myocardial apoptosis in diabetic mice [28] and improved learning and memory ability in mice following acute alcohol intoxication [10] through its antioxidant properties in previous studies. Here, we provided additional evidence that FPE prevents cognitive impairment in experimental diabetic mice. In contrast to the kept increasing large population of diabetes and AD group, effective intervention in clinic is very limited and bioactive compounds from natural plant may serve as the leads or scaffolds for further chemical elaboration. Thus, FPE could be a promising candidate for therapy associated with diabetes and dementia.

Taken together, in the present study we have demonstrated that FPE treatment could significantly normalize metabolic disorder and improve cognitive deficits in STZ-induced experimental diabetic mice, and this effect is likely related to inhibition oxidative stress and AChE activity in brain.
